# Prevalence of soil-transmitted helminths infections among preschool and school-age children in Ethiopia: a systematic review and meta-analysis

**DOI:** 10.1186/s41256-022-00239-1

**Published:** 2022-03-21

**Authors:** Legese Chelkeba, Zeleke Mekonnen, Daniel Emana, Worku Jimma, Tsegaye Melaku

**Affiliations:** 1 Department of Pharmacology and Clinical Pharmacy, School of Pharmacy, College Health Sciences, Black Lion Specialized Hospital, Addis Ababa University, Addis Ababa, Ethiopia; 2 Department of Parasitology, School of Medical Laboratory Sciences, Institute of Health, Jimma University, Jimma, Ethiopia; 3 Department of Information Science, College of Natural Sciences, Jimma University, Jimma, Ethiopia; 4 Department of Clinical Pharmacy, School of Pharmacy, Institute of Health, Jimma University, Jimma, Ethiopia

**Keywords:** Soil-transmitted helminths, Preschool-age children, School-age children, Meta-analysis, Ethiopia

## Abstract

**Background:**

There is a lack of comprehensive national data on prevalence, geographical distribution of different species, and temporal trends in soil-helminthiasis (STHs). Therefore, this study aimed to provide a summary and location of the available data on STHs infection among preschool and school-age children in Ethiopia.

**Methods:**

The search was carried out in Medline via PubMed, Scopus, Science Direct, Web of Science, and Google Scholar on data published between 1997 to February 2020 for studies describing the rate of STHs infection among preschool and school-age in Ethiopian. We followed the Patient, Intervention, Comparison, and Outcome (PICO) approach to identify the studies. Meta-regression was performed to understand the trends and to summarize the prevalence using the “metaprop” command using STATA software version 14.0

**Results:**

A total of 29,311 of the 61,690 children examined during the period under review were infected with one or more species of intestinal parasites yielding an overall prevalence of 48% (95% CI: 43–53%). The overall pooled estimate of STHs was 33% (95% CI: 28–38%). The prevalence was 44% (95% CI: 31–58%) in SNNPR, 34% (95% CI: 28–41%) in Amhara region, 31% (95% CI: 19–43%) in Oromia region and 10% (95% CI: 7–12%) in Tigray region. Soil-transmitted helminths infection rate has been decreasing from 44% (95% CI: 30–57%) pre-Mass Drug Administration (MDA) era (1997–2012) to 30% (95% CI: 25–34%) post-MDA (2013–2020), although statistically not significant (*p* = 0.45). *A lumbricoides* was the predominant species with a prevalence of 17%.

**Conclusion:**

Southern Nations Nationalities and Peoples Region, Amhara, and Oromia regions carry the highest-burden and are categorized to Moderate Risk Zones (MRZ) and therefore, requiring MDA once annually with Albendazole or Mebendazole. The prevalence of STHs decreased after MDA compared to before MDA, but the decline was not statistically significant. *A. lumbricoides* was the predominant species of STHs among preschool and school-age children in Ethiopia. The high prevalence of STHs observed in this review, underscores the need for better control and prevention strategies in Ethiopia.

**Supplementary Information:**

The online version contains supplementary material available at 10.1186/s41256-022-00239-1.

## Background

Soil-transmitted helminths (STHs) infections are one of the most common infections in countries with limited resources. Globally, more than 4.5 billion people are at risk of infection and nearly 2 billion are infected with STHs [1, 2]. In contrast to other infectious diseases, infection due to STHs such as *Ascaris lumbricoides,* hookworm species*, and Trichuris trichiura* do not usually cause significant mortality rates; instead adapted to chronic illness and extended morbidity affecting poor people [3–6].

Transmissions of the STHs are mainly by eggs or larvae that are passed with feces of an infected person or hatched in the soil after defecation. Adult worms residing in the gut of an infected person produce thousands of eggs every day, which may contaminate environments or foods that lack adequate sanitation [7]. Additionally, climatic conditions of tropical and sub-tropical countries are suitable for the survival of STH eggs and larvae hatching and embryonated in warm temperature and adequate moisture soil [8]. Consequently, the complication of STH may cause gut blood losses, malabsorption of nutrients, loss of appetites, and anemia due to loss of iron and other important protein [9]. For instance, the outcome of the infection on the children results in serious problems such as anemia, growth retardation, impaired cognitive developments, school absenteeism, and disability-adjusted life-years lost [10, 11].

World Health Organization (WHO) has published comprehensive road map data in 2012 to combat Neglected tropical diseases (NTDs) by 2020. Mass Drug Administration (MDA) approach was also designed to undertake 75% coverage in all of the known endemic countries for STHs. The ideas of WHO was strengthened by the London declaration to control or eliminate other ten [10) NTDs in addition to the STHs [12, 13]. Recently, following WHO strategic plan, Ethiopia has launched a nationwide MDA to control STHs, which targets 17 million children within the age range of 5–14 years old. The Ethiopian Ministry of Health and WHO started deworming in 2013, of which approximately 6.8 million and 7.8 million pre-school children (PSAC) and school-age children (SAC) were treated, respectively. Even before the deworming program, the ministry of health has undertaken some other measures to control poverty-related diseases including STHs among the population at risk, particularly SAC. For instance, the implementation of a health extension program focusing on creating awareness on latrine construction and utilization and keeping personal and environmental hygiene among the community is one priority program since 2003/2004. However, current individual reports indicated that the prevalence of STHs in Ethiopia is not declining. A large-scale study conducted in Amhara regional state showed that the prevalence of STHs was 36.4% [14]. Another study in Jimma town showed that the prevalence of STHs among SAC was 49.0% [15]. A similar study also reported that the prevalence of STHs was 47% in the rural community of Ethiopia [16]. Nevertheless, numerous fragmented studies have been carried out on assessing the prevalence of STHs among preschool children (PSAC) and SAC in Ethiopia, but comprehensive nationwide data on the prevalence, geographic distribution of different species, and time trends of STHs are lacking. Therefore, this study aimed to provide a summary of prevalence, geographical location, and time trends of STHs among preschool and SAC to measure the impact of the ongoing control and preventive measures in the country. In addition, such an effort might help the government and other concerned bodies to focus on specific areas of high prevalence for further preventive measures such as chemotherapy and improved sanitation practices.

## Methods

We used and were guided by PRISMA (Preferred Reporting Items for Systematic Reviews and Meta-Analyses) [17] guideline and checklist to carry out the current Systematic Reviews and Meta-analyses. The outcome of interest was the prevalence of STHs among PSAC and SAC in Ethiopia.

### Search strategy

The search was carried out in Medline via PubMed, Scopus, Science Direct, Web of Science, and Google Scholar using searching terms such as **“**intestinal helminths’’, ‘’intestinal parasites’’, "soil-transmitted helminths”, ‘’STHs’’, ‘’ *Strongyloides stercoralis* '', ‘’*Ascaris lumbricoides*’’, ‘’ *Trichuris trichiura*'', '' Hookworms’’, ‘’preschool-age’’, ‘’school-age’’, “Ethiopia”. These key terms were combined using "AND" and "OR" Boolean operators. Medical Subject Headings (MeSH terms) were used to search relevant original articles in PubMed. Searching was carried out on articles published between 1997 to February 2020 and limited to English language and human studies. A manual search for additional relevant studies using references from retrieved articles and related systematic reviews was also performed to identify original articles we might have missed.

Endnote citation manager software version X_9_ for Windows was utilized to collect and organize search outcomes (into relevant and irrelevant studies) and for the removal of duplicate articles. We followed the PICO approach to identify the relevant articles:

- Population (P): School-age children
- Exposure (E): Presence of soil-transmitted helminths
- Comparison (C): Preschool-age children
- Outcome (O): Prevalence of soil-transmitted helminths.

**Prevalence** was calculated as the number of subjects positive for STHs in the study divided by the total number of participants in a study multiplied by 100.

### Inclusion and exclusion criteria

We included observational studies conducted between 1997 to February 2020 which documented the baseline prevalence or incidence of STHs and studies published in the English language targeting both PSAC (< 5 years) and SAC (≥ 5 years). We excluded case reports, case series, studies that compared the sensitivity and specificity of different methods for diagnosis of STHs, and studies not reported either prevalence or incidence as an outcome of interest. This is due to those articles and/or reports will not adequately address the review objective. The current review didn’t include unpublished studies or grey literature.

### Data abstraction and quality assessment

Following preliminary assessment and downloading of the abstracts by two authors, they were assessed for agreement with the inclusion criteria. Irrelevant articles (articles that were out of the scope of the study) were excluded after assessment of the abstracts unless it was unclear to classify articles into irrelevant based on abstracts, where we downloaded the full text for further clarity. Once articles were deemed to be relevant, the full text of the articles was downloaded for further detailed review. We extracted information on the name of the first author and year of publication, study design, gender, region of study, laboratory method identification of the parasites, total sample size, the number of positives for intestinal parasites in general, number of positive for STHs in particular, and quality score for quality assessment. The Grading of Recommendation Assessment, development, and Evaluation (GRADE) approach was used to assess the overall quality of evidence [18]. Studies were given one point each if they had probability sampling, larger sample sizes of more than 200, and repeated detection, and up to four points could be assigned to each study. We regarded publications with a total score of 3–4 points to be of high quality, whereas 2 points represented moderate quality and scores of 0–1 represented low quality.

### Statistical analysis

We used forest plots to estimate pooled effect size and effect of each study with their confidence interval (CI) to provide a visual summary of the data. A random-effects model was used in this meta-analysis because of anticipated heterogeneity. Statistical heterogeneity among studies was expressed as the Cochrane’s Q test and I^2^, where a *p* < 0.05 and I^2^ values of 0, 25, 50, and 75% were considered as no, low, moderate, and high heterogeneities, respectively. Because we expected geographical variation and socio-economic contexts might differ radically across these studies, subgroup analysis based on the geography of the region, age children included, and year of publication. In addition to visual inspection for symmetry of the plot, we also used Begg's Funnel plot and Egger's regression test for quantitative evaluation of the possibility of publication bias. Meta-regression analysis was employed to identify the source of heterogeneity using regional states, age of children, publication years, and study design as covariates. All reported *p* values were 2-sided and were statistically significant if *p* < 0.05.

## Results

### Literature searches and selection

**Fig. 1:**
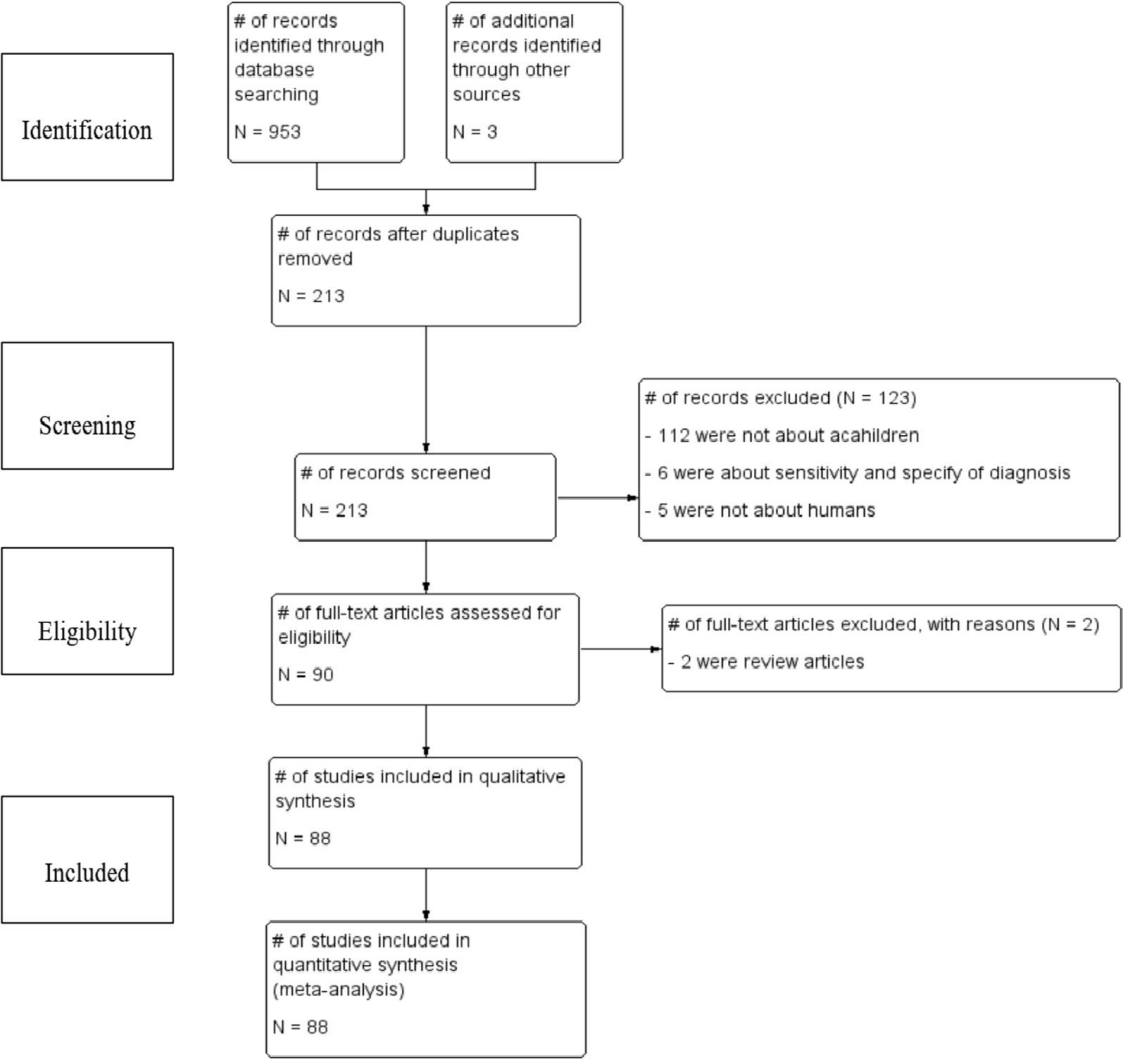
Flow diagram showing the selection process

Our initial search of electronic databases such as Medline via PubMed, Scopus, Science Direct, Web of Sciences, and Google scholar yielded 953 articles and 3 articles manually from which 213 records remained after removing duplications. Upon screening the articles, 123 articles were further excluded; 112 were irrelevant because they were not specifically about PSACor SAC, 6 studies were about sensitivity and specificity of diagnosis of STHs, and 5 articles were not about humans. Upon further assessment for eligibility, 2 studies were excluded being review articles. Finally, 88 [6, 14, 16, 19–110] published studies between 1997 and February 2020 fulfilling the inclusion criteria were included in the final analyses (Fig. 1). The sample size of the included studies ranged from 100 [20] to 15,455 [14]. A total of 61,690 children with age of < 5 years (n = 5577) and ≥ 5 years (n = 55,731) or mix of both (n = 382) were recruited in the studies. Fifty-two percent (52%) of the study participants were male. The majority (83) of the studies were cross-sectional. Seventy-three studies were about STHs in SAC, thirteen were about preschool-age and the rest were studies that involved both PSAC and SAC. Thirty-five and twenty-four studies used Kato-Katz or in combination with other tools and formalin-ether concentration plus direct microscopic method for screening stools, respectively. Formalin-ether concentration techniques in 19 studies, direct wet mount method in 5 studies, McMaster in 4 studies, and Harada Mori (Test tube culture) technique in one study utilized as screening of stools. According to our quality assessment criteria, 43 publications were of high quality with a score of 3; 11 had a score of 2 indicating moderate quality; and the remaining 34 were of low quality with a score of zero or one (Table 1).

**Table 1:** Characteristics of the eligible studies on STH infections in Ethiopia

References	Study design	Population	Male	Female	Region	Laboratory methods	No. sample	Cases for IPIs	Quality score
Mekonnen et al. [6]	Clinical trial	School children	NA	NA	Oromia	KATO	840	421	3
Nute et al. [14]	Cross-sectional	School children	7418	8037	Amhara	FEC	15455	5626	3
Gadisa et al [16]	Cross sectional	Preschool children	242	319	Oromia	DWM&FEC	561	216	3
Alamir et al. [19]	Cross sectional	School children	192	207	Amhara	DWM&FEC	399	104	0
Fekadu et al. [20]	Cross sectional	School children	63	37	Oromia	Harada-Mori (Test tube culture)	100	470	0
Dessalegn et al. [21]	Cross-sectional	School children	271	315	Oromia	DWM&FEC	586	91	3
Fentie et al. [22]	Cross-sectional	School children	361	159	Amhara	KATO&FEC	520	134	3
Haileamlak et al. [23]	Cross sectional	Preschool children	487	437	Oromia	DWM&FEC	924	74	1
Mulatu et al. [24]	Cross-sectional	Preschool	81	77	SNNPR	DWM,FEC&MZN	158	224	3
Alemu et al. [25]	Cross sectional	School children	157	162	Amhara	KATO&DWM	319	243	3
Amare et al. [26]	Cross-sectional	School children	218	187	Amhara	KTO &FEC	405	92	3
Ayalew et al. [27]	Cross sectional	School children	358	346	Amhara	DWM&FEC	704	304	2
Beyene et al. [28]	Cross sectional	School children	114	146	Oromia	DWM&FEC	260	328	3
Merid et al. [30]	Cross sectional	School children	NA	NA	SNNPR	DWM&FEC	150	465	0
Nguyen et al. [31]	Cross sectional	School children	341	323	Amhara	FEC	664	129	3
Nyantekyi et al. [32]	Cross sectional	Preschool children	140	148	SNNPR	KATO&FEC	288	282	1
Tadesse et al. [33]	Cross sectional	School children	271	144	Oromia	FEC	415	437	0
Degarege et al. [34]	Cross-sectional	School children	187	216	Amhara	KATO	403	255	2
Jejaw et al. [36]	Cross-sectional	School children	228	232	SNNPR	DWM,FEC&KATO	460	353	3
King et al. [37]	cross sectional	Both	1130	1228	Amhara	FEC	2,338	267	3
Tulu et al. [38]	Cross sectional	School children	172	168	SNNPR	DWM&FEC	340	113	1
Erosie et al. [40]	Cross sectional	School children	NA	NA	SNNPR	FEC	421	69	1
Gelaw et al. [41]	cross-sectional	School children	170	134	Amhara	DWM&FEC	304	104	3
Jemaneh et al. [42]	Cross sectional	School children	282	405	Amhara	KATO	687	219	1
Mahmud et al. [43]	cross-sectional	School children	288	312	Tigray	DWM,FEC&KATO	600	89	3
Mahmud et al. [44]	Clinical trial	School children	152	217	Tigray	DWM,FEC&KATO	369	326	3
Reji et al. [45]	Cross sectional	School children	NA	NA	Oromia	KATO	358	52	1
Roma et al. [46]	Cross sectional	School children	352	168	SNNPR	FEC	520	233	1
Adamu et al. [48]	Cross sectional	Preschool children	149	147	Addis Ababa	DWM,FEC&MZN	296	571	0
Belyhun et al. [49]	Follow up cohort	Preschool children	NA	NA	SNNPR	FEC	905	292	3
Legesse et al [52]	Cross sectional	School children	167	214	Oromia	KATO&FEC	381	166	0
Mathewos et al. [53]	Cross-sectional	School children	139	122	Amhara	DWM&MZN	261	174	2
Alemayehu et al. [54]	Cross-sectional	School children	287	216	SNNPR	KATO&FEC	503	363	3
Dejenie et al. [56]	Cross sectional	School children	1012	998	Tigray	DWM	2000	245	0
Gashaw et al. [57]	Cross-sectional	School children	255	295	Amhara	KATO	550	365	3
Tekeste et al. [60]	Cross sectional	School children	170	156	Amhara	KATO	326	109	2
Terefe et al. [61]	Cross sectional	School children	218	201	SNNPR	KATO	419	285	1
Wale et al. [62]	Cross sectional	School children	206	196	Amhara	DWM&FEC	402	562	1
Aiemjoy et al. [63]	Cross-sectional	Preschool children	NA	NA	Amhara	FEC	212	354	2
Alemu et al. [64]	Cross sectional	School children	211	194	SNNPR	KATO	405	110	0
Tullu et al. [65]	Cross sectional	School children	251	241	Oromia	DWM&FEC	492	44	0
Dejenie et al. [66]	Cross sectional	School children	319	303	Tigray	KATO	622	263	0
Teklemariam et al. [67]	Cross sectional	School children	252	228	Tigray	FEC	480	139	0
Zemene et al. [68]	Cross-sectional	Preschool children	118	118	Amhara	DWM&FEC	247	43	1
Firdu et al. [69]	Case-control	Both	135	95	SNNPR	DWM,FEC&MZN	230	199	1
Tefera et al. [70]	Cross sectional	School children	282	433	Oromia	McMaster	715	202	2
Unasho et al. [71]	Cross sectional	School children	189	217	SNNPR	DWM	406	89	0
Yimam et al. [72]	Cross-sectional	School children	187	216	Amhara	KTO&FEC	403	235	3
Abdi et al. [73]	cross-sectional	School children	207	201	Amhara	FEC	408	282	3
Abera et al. [74]	Cross-sectional	School children	193	192	Amhara	FEC	385	357	3
Abossie et al. [76]	Cross-sectional	School children	191	209	SNNPR	DWM&FEC	400	324	3
Hailegebriel et al. [77]	Cross-sectional	School children	177	182	Amhara	FEC	359	235	3
Alemu et al. [78]	Cross-sectional	School children	196	195	SNNPR	FCE	391	182	2
Alemu, et al. [79]	Cross- sectional	Preschool children	183	218	Amhara	KATO	401	141	3
Alemu et al. [80]	Cross-sectional	School children	180	171	SNNPR	DWM&FEC	351	95	3
Amor et al. [82]	Cross-sectional	School children	225	171	Amhara	FEC	396	327	3
Bekana et al. [84]	Cross-sectional	School children	172	145	Oromia	KATO&FEC	317	130	3
Diro et al. [85]	prospective cohort	Both	85	37	Amhara	DWM,FEC&KATO	122	371	1
Birhanu et al. [86]	cross sectional	School children	194	228	Benishangul-Gumuz	DWM	422	138	1
Leta et al. [87]	Cross sectional	School children	NA	NA	Amhara	KATO	2,650	437	3
Tefera et al. [88]	Cross sectional	School children	364	280	Oromia	McMaster	644	237	2
Alemayehu et al. [90]	Cross sectional	School children	201	183	SNNPR	KATO&DWM	384	131	1
Ali et al. [91]	Cross sectional	School children	161	121	Oromia	KATO&DWM	282	170	0
Jemaneh et al. [92]	Cross sectional	School children	439	439	Amhara	KATO	878	165	0
Debalke et al. [93]	Cross sectional	School children	161	205	Oromia	McMaster	366	66	1
Dejene et al. [94]	Cross sectional	School children	481	319	Tigray	FEC	800	530	0
Abera et al. [95]	cross sectional	School children	397	381	Amhara	KATO&FEC	772	311	3
Kabeta et al. [96]	Cross sectional	Preschool children	NA	NA	SNNPR	DWM&FEC	587	254	1
Shumbej et al. [97]	Cross sectional	Preschool children	165	212	SNNPR	McMaster	377	245	3
Tadege et al [98]	Cross sectional	School children	235	139	SNNPR	FEC	374	127	3
Andualem et al. [99]	Cross sectional	School children	168	190	Amhara	DWM&FEC	358	59	0
Teshale et al. [100]	Cross section	School children	240	170	Tigray	KATO	410	58	1
Elfu et al. [101]	Cross sectional	School children	1129	1261	Amhara	DWM&FEC	2390	684	3
Eyamo et al. [102]	Cross sectional	School children	199	185	SNNPR	DWM	384	260	3
Molla et al. [104]	Cross sectional	School children	245	198	SNNPR	KATO	443	239	3
Tadesse et al. [106]	Cross sectional	School children	204	213	Oromia	DWM&FEC	422	131	2
Weldesenbet et al. [107]	Cross sectional	School children	349	251	SNNPR	KATO	600	57	3
Workineh et al. [108]	Cross sectional	School children	194	146	Amhara	KATO	340	51	3
Shumbej et al. [109]	Cross sectional	School children	350	247	SNNPR	KATO	597	141	3
Zenu et al. [110]	Cross sectional	School children	284	28	Oromia	DWM&FEC	312	208	3
Gizaw et al. [111]	Cross-sectional	Preschool children	106	119	Amhara	KATO	225	58	3
Mekonnen et al. [112]	Cross-sectional	Preschool children	152	158	Amhara	DWM&KATO	310	58	3
Gebrehiwot et al. [113]	Cross sectional	Preschool children	195	179	Oromia	KATO	374	1471	2
Hailu et al. [114]	Cross sectional	School children	186	223	Amhara	Richie’s	409	263	2
Assefa et al. [115]	Cross sectional	School children	479	219	Amhara	FEC	698	401	0
Kidane et al. [116]	Cross sectional	School children	177	207	Tigray	DWM	384	301	0
Samuel et al. [117]	Cross sectional	School children	NA	NA	Oromia	FEC	375	42	3

*DWM* direct wet mount; *FEC* formal-ether, *KATO* kato-katz, *NNNPR* Southern nations nationalities and peoples region, *IPIs* intestinal parasitic infections, *STHs* soil transmitted helminthes, *NA* not available

### The pooled prevalence estimate of intestinal parasites and heterogeneity

**Fig. 2:**
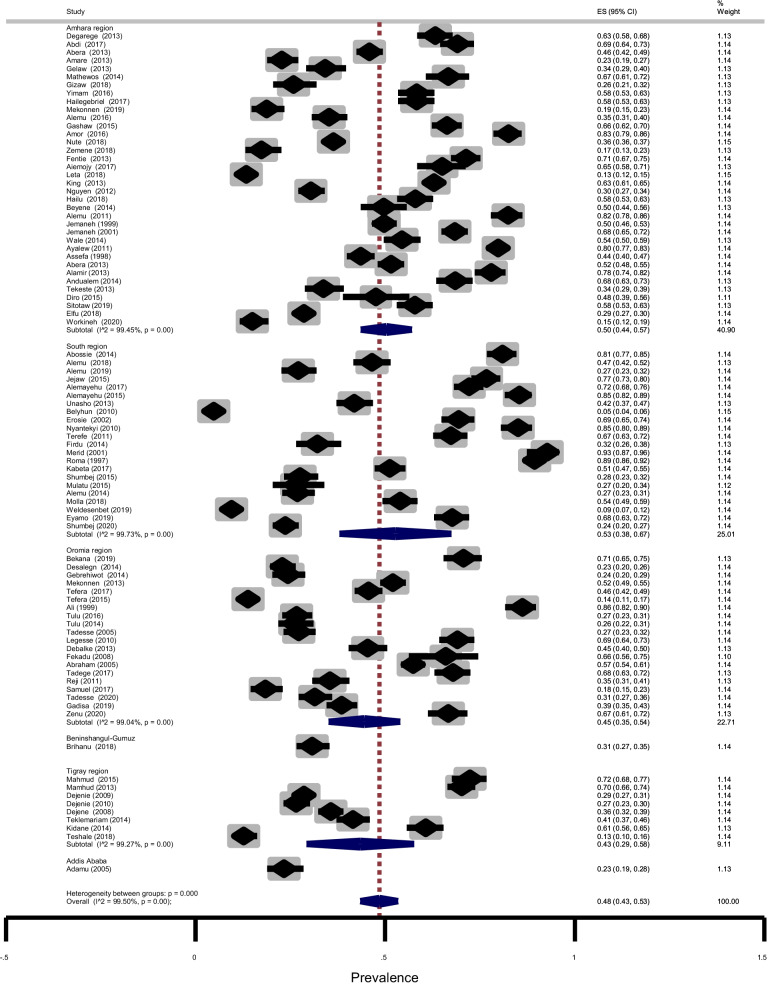
Forest plot showing pooled prevalence of intestinal parasites among children according to regional states in Ethiopia

Eighty studies (88) studies consisting of 61,690 PSAC and SAC reported the proportion of intestinal parasitic infections. Out of these, 29,311 children were infected with one or more species of intestinal parasites giving the pooled prevalence estimate of 48% (95% CI: 43–53%) with considerable heterogeneity (χ^2^ = 17,303.64, *p* < 0.001; I^2^ = 99.50%). The prevalence of intestinal parasitic infection was 53% (95% CI: 38–67%), 50% (95% CI: 44–57%), 45% (95% CI: 35–54%) and 43% (95% CI: 29–58%) in Southern Nations Nationalities and Peoples Region (SNNPR), Amhara, Oromia, and Tigray regions, respectively (Fig. 2). We also did a subgroup analysis to see the influence of study design on prevalence. Interestingly enough, the prevalence was 48% (95% CI: 43–53%) for cross-sectional study design and therefore, the inclusion of other study designs does not influence the overall rate of infection (not shown).

### The overall prevalence estimate of soil-transmitted helminths (STHs) and heterogeneity

**Fig. 3:**
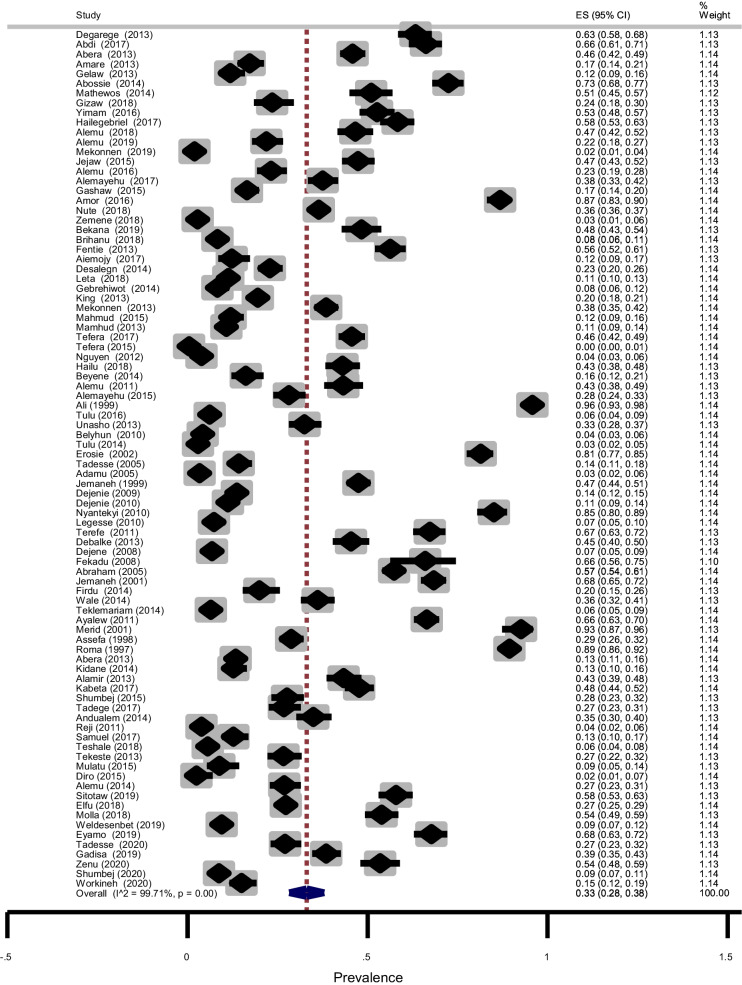
Forest plot showing pooled prevalence of STHs

**Fig. 4:**
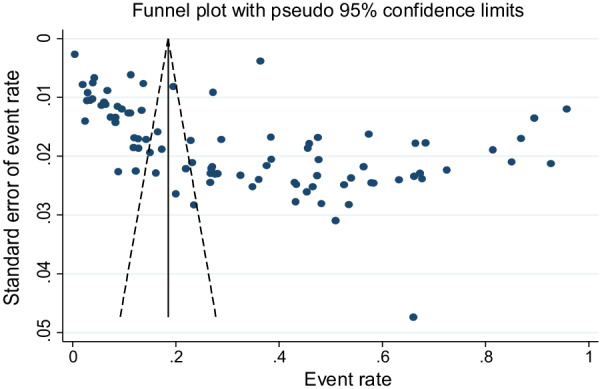
Publication bias assessment plot: Egger’s regression test (*p* < 0.0001) and Begg’s rank correlation (*p* < 0.001)

Soil-transmitted helminths detected in the studies were *Ascaris lumbricoides*, Hookworms, *Trichuris trichiura, and Strongyloides stercoralis.* A total of 19, 678 of the 61,690 children examined during the period under review were infected with one or more species of STHs yielding an overall prevalence of 33% (95% CI: 28–38%) with substantial heterogeneity (χ^2^ = 30,360.02, *p* < 0.001; I^2^ = 99.71%) (Fig. 3). The asymmetry of funnel plot visual inspection (Fig. 4) showed that the presence of publication bias which was statistically confirmed by Egger’s test (β = 16.7, [95% CI: 10.7–22.5]), *p* < 0.001 and Begg’s test *p* < 0.001.

**Fig. 5:**
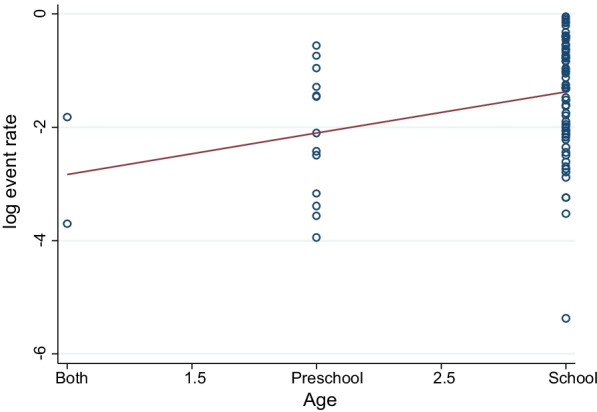
Meta-regression of prevalence of STHs (log event rate) by Age

We did meta-regression analyses to search for the sources of heterogeneity. A univariate meta-regression between the prevalence of STHs and the age of children showed a statistically significant correlation (*p* = 0.003, Fig. 5). However, year of publications, (*p* = 0.076), regional states (*p* = 0.70) and study design (*p* = 0.23) did not show a statistically significant correlation as shown in Table 2.

**Table 2:** Univariate meta-regression of factors related to the heterogeneity of soil-transmitted helminths among Ethiopian children, 2020

Variables	β-coefficient	95% CI	*p* values
Regional states	− 0.03	− 0.18 to 0.12	0.70
Year of publication	− 0.04	− 0.004 to 0.08	0.076
Age	0.73	0.25 to 1.2	0.003
Study design	− 0.45	− 1.2 to 0.30	0.23

*CI* confidence interval

### Sub-group analysis based on geographical region and age of children

**Fig. 6:**
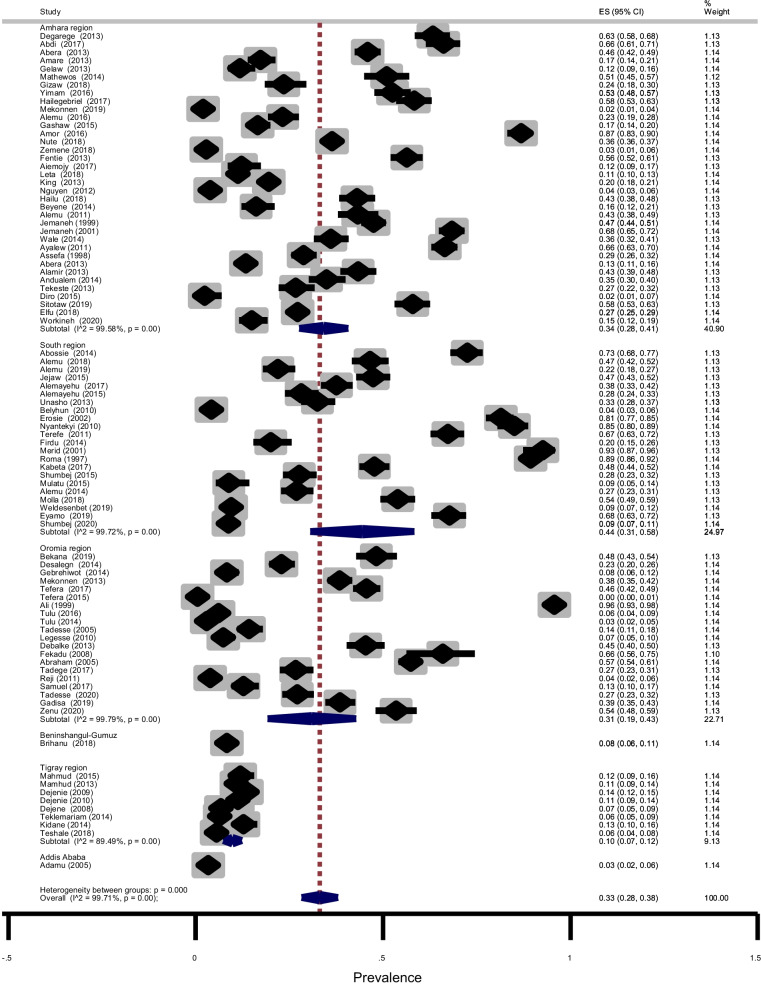
Forest plot showing prevalence of STHs by region

**Fig. 7:**
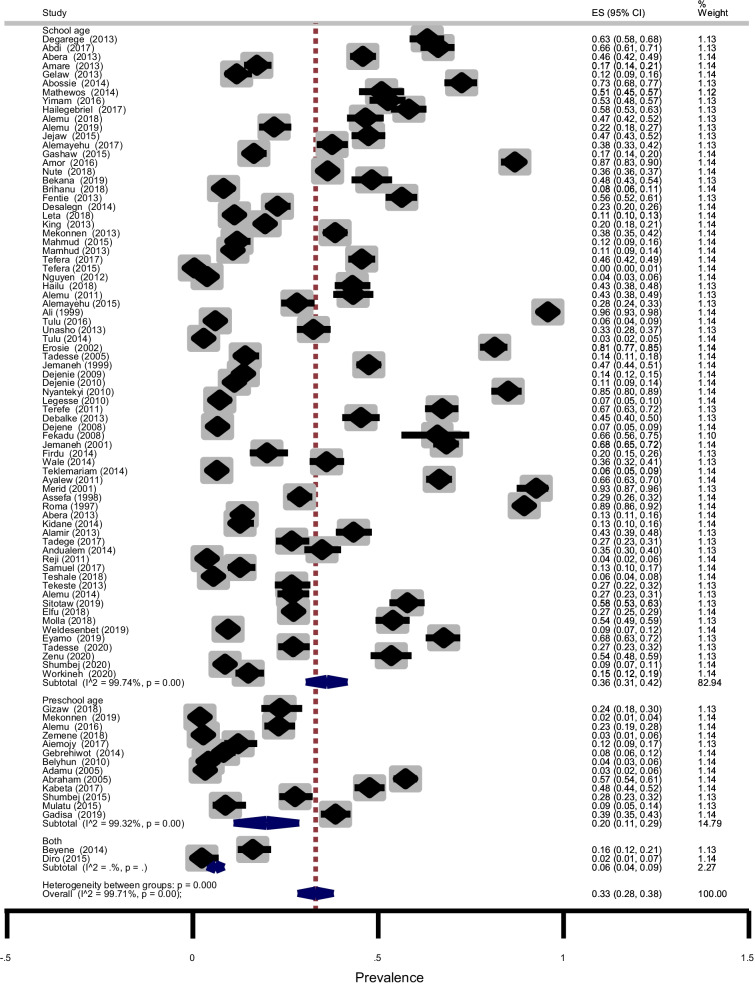
Forest plot showing prevalence of STHs by age

**Fig. 8:**
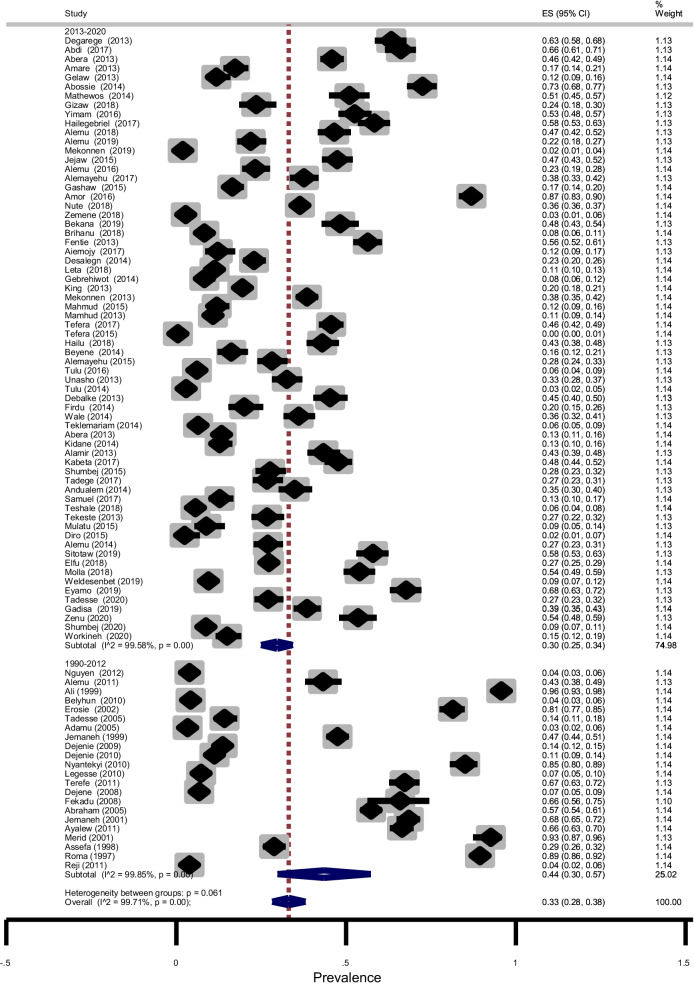
Forest plot showing prevalence of STHs by year of publication

Subgroup analysis showed that the prevalence of STHs was 44% (95% CI: 31–58%) in SNNPR, 34%(95% CI: 28–41%) in Amhara region, 31% (95% CI: 19–43%) in Oromia region and 10% (95% CI: 7–12%) in Tigray region as shown in Fig. 6. The age-related prevalence was 51% (95% CI: 45–56%) in SAC and 32% (95% CI: 20–44%) in PSAC (*p* = 0.003) as shown in Fig. 7. Subgroup analysis by publication year showed that the pooled prevalence of STHs between 1995 and 2012 years was 44% (95% CI: 30–57%) while, it was 30% (95% CI: 25–34%) for studies conducted between 2013 and 2020 years (Fig. 8). In summary, STHs were more common in SNNPR among SAC in studies published between 1990 and 2012 as shown in Table 3. We performed subgroup analysis based on study design and the result showed that the prevalence of STHs was 34% (95% CI: 29–39%) for cross-sectional study, 25% (95% CI: 23–28%), 4% (95% CI: 3–5%) for prospective study and 20% (95% CI: 15–26%) for case–control study (not shown). This indicates that the overall prevalence is almost the same as the prevalence of studies with cross-sectional study design and was not affected by other study designs.

**Table 3:** Prevalence of soil-transmitted helminths (STHs) by region, age of children and year of publication among Ethiopian Children, 2020

Variables	No. of studies	Sample	Cases	Prevalence (95% CI)	Heterogeneity		*p* value
					Q	I^2^ (%)	
*Region*							
Addis Ababa city	1	296	10	3 (2–6%)	–	–	–
Amhara region	36	36,809	12,374	34 (28–41%)	8325.55	99.58	*p* < 0.001
Benishangul-Gumuz region	1	422	35	8 (6–11%)	–	–	–
Oromia region	20	9119	2780	31 (19–43%)	9070.41	99.79	*p* < 0.001
South Region	22	9379	3869	44 (31–58%)	7621.83	99.72	*p* < 0.001
Tigray region	8	5665	610	10 (7–12%	66.61	89.49	*p* < 0.001
*Age*							
School	73	55,731	18,225	36 (31–42%)	27,820.22	99.74	*p* < 0.001
Preschool	13	5577	1408	20 (11–29%)	1764.59	99.32	*p* < 0.001
Both	2	382	45	6 (4–9%)	–	–	–
*Year of publication*							
1997–2012	22	12,831	4607	44 (30–57%)	14,221.00	99.85	*p* < 0.001
2013–2020	66	48,859	15,071	30 (25–34%)	15,324.02	99.58	*p* < 0.001
Overall	88	61,690	19,678	33 (28–38%)	30,360.02	99.71	*p* < 0.001

*CI* Confidence Interval

### Prevalence of STHs by species

#### Ascaris lumbricoides

Eighty five studies consisting of 58, 234 children have reported that the pooled prevalence of *A. lumbricoides* was 17% (95% CI: 15–19%) with substantial heterogeneity (χ2 = 8961.94, *p* < 0.001; I^2^ = 99.06%). The prevalence was 27% (95% CI: 21–34%) in SNNPR, 14% (95% CI: 11–17%) in Amhara region, 15% (95% CI: 11–19%) in Oromia region and 6% (95% CI: 3–8%) in Tigray region (Additional file 1: Fig. S1). The age related prevalence of *A. lumbricoides* was 18% (95% CI: 15–20%) in SAC and 12% (95% CI: 8–17%) in PSAC (*p* = 0.06). The pooled prevalence of *A. lumbricoides* was 25% (95% CI: 19–31%) in studies published between 1997 and 2012 years and 14% (95% CI: 12–16%) between 2013 and 2020 years. A univariate meta-regression between prevalence and year of publications showed statistically significant correlation [β = −0.49 (95% CI: −1.1 to −0.07, *p* = 0.035)] (Additional file 1: Fig. S2). However, regional states [β: 0.046, (95% CI: −0.12 to 0. 0.22, *p* = 0.58)] and age of children [β: 0.52, (95% CI: −0.02 to 1.1, *p* = 0.06)] did not show a statistically significant relationship. Therefore, *Ascaris lumbricoides* was the most predominant species of STHs among Ethiopian children and significant decline in prevalence was observed over two decades (from late 1990s to 2020) (Table 4).

**Table 4:** Pooled prevalence of species specific *Ascaris lumbricoides* by region, age and year of publication among Ethiopian children, 2020

Variables	No. of studies	Sample	cases	Prevalence (95% CI)	Heterogeneity		*p* value
					Q	I^2^ (%)	
*Region*							
Addis Ababa city	1	296	8	3 (1–5%)	–	–	–
Amhara region	35	34,419	5311	14 (11–17%)	3356.20	98.99	*p* < 0.001
Oromia region	19	8475	1271	15 (11–19%)	934.49	98.07	*p* < 0.001
South Region	22	9379	2374	27 (21–34%)	4265.90	99.51	*p* < 0.001
Tigray region	8	5665	375	6 (3–8%)	148.81	95.30	*p* < 0.001
*Age*							
School	70	52,275	8509	18 (15–20%)	7820.37	99.12	*p* < 0.001
Preschool	13	5577	822	12 (8–17%)	892.82	98.66	*p* < 0.001
Both	2	382	8	2 (1–4%)	–	–	–
*Year of publication*							
1990–2012	22	12,831	2841	25 (19–31%)	4111.93	99.49	*p* < 0.001
2013–2020	63	45,403	6498	14 (12–16%)	4838.10	98.72	*p* < 0.001
Overall	85	58,234	9339	17 (15–19%)	8961.94	99.06	*p* < 0.001

*CI* Confidence Interval

#### Trichuris trichiura

Seventy six studies included of 54,854 children have reported that the pooled prevalence of ***Trichuris trichiura*** was 6% (95% CI: 6–7%) with considerable heterogeneity (χ2 = 3766.86, *p* < 0.001; I^2^ = 98.01%). The pooled prevalence was 11% (95% CI: 11–13%) in SNNPR, 10% (95% CI: 8–13%) in Oromia region, 4% (95% CI: 3–4%) in Amhara region and 1% (95% CI: 0–2%) in Tigray region and 1 (Additional file 1: Fig. S3). The age related prevalence was also 7% (95% CI: 6–8%,) among SAC and 4% (95% CI: 2–6%) among PSAC (*p* = 0.24). The pooled prevalence of *T. trichura* was 14% (95% CI: 12–17%) in studies conducted between 1997 and 2013 years and 4% (95% CI: 4–24%) between 2013 and 2020 years. A univariate meta-regression between prevalence and year of publications showed statistically significant correlation [Β = − 0.78, (95% CI: − 1.5 to − 0.069, *p* = 0.03)] (Additional file 1: Fig. S4). However, regional states [β: 0.003, (95% CI: − 0.22 to 0. 0.23, *p* = 0.97)] and age of children [β: 0.46 (95% CI: − 0.29 to 1.2, *p* = 0.23)] did not show a statistically significant relationship. The bottom line is that the rate of infection of *Trichuris trichiura* among Ethiopian children decreased significantly after starting of MDA as detailed in Fig. 5*.* In addition, infection from Trichuris trichiura was more prevalent among children in SNNP region and Oromia region compared to other regions and also significant decline in prevalence was observed over two decades (Table 5).

**Table 5:** Pooled prevalence of species specific *Trichuris trichiura* by region, age and year of publication among Ethiopian children, 2020

Variables	No. of studies	Sample	cases	Prevalence (95% CI)	Heterogeneity		*p *value
					Q	I^2^ (%)	
Region							
Addis Ababa city	1	296	2	1 (0–2%)	–	–	–
Amhara region	28	31,555	1186	4 (3–4%)	528.28	94.89	*p* < 0.001
Oromia region	19	8738	1089	10 (8–13%)	1282.36	98.60	*p* < 0.001
South Region	22	9379	949	11 (9–13%)	1728.08	98.78	*p* < 0.001
Tigray region	6	4886	86	1 (0–2%)	61.86	91.92	*p* < 0.001
Age							
School	63	49,327	3093	7 (6–8%)	3318.18	98.13	*p* < 0.001
Preschool	12	5267	211	4 (2–6%)	443.50	97.52	*p* < 0.001
Both	1	260	8	3 (2–6%)	–	–	–
Year of publication							
1990–2012	20	11,786	1374	14 (12–17%)	2176.26	99.13	*p* < 0.001
2013–2020	56	43,068	1938	4 (4–5%)	1569.21	96.50	*p* < 0.001
Overall	76	58,234	9339	6 (6–7%)	3766.86	98.01	*p* < 0.001

*CI* Confidence interval

#### Hookworms

Seventy six studies consisting of 54,854 children have also reported the pooled prevalence of Hookworms. Hence, the pooled prevalence on analysis was 12% (95% CI: 10–13%) with substantial heterogeneity (χ2 = 7920.16, *p* < 0.001; I^2^ = 99.05%). The pooled prevalence of hookworms was 12% (95% CI: 9–15%) in SNNPR, 16% (95% CI: 13–19%) in Amhara region, 6% (95% CI: 5–8%) in Oromia region, and 3% (95% CI: 2–4%) in Tigray region as shown in Additional file 1: Fig. S5. The age related prevalence of hookworms was 13% (95% CI: 11–15%) among SAC and 2% (95% CI: 1–3%) among PSAC (*p* = 0.01). The pooled prevalence of hookworms was 13% (95% CI: 9–15%) in studied conducted between 1997 and 2012 years and 11% (95% CI: 9–13%) in studies between 2013 and 2020 years.

A univariate meta-regression between prevalence and age of children showed statistically significant correlation [Β = 1.03, (95% CI: 0.27–1.8, *p* = 0.01)], (Additional file 1: Fig. S6A). Additionally, meta-regression of the prevalence and regional states [β: − 0.20, (95% CI: − 0.40 to − 0. 0.005, *p* = 0.045)], (Additional file 1: Fig. S6B) revealed a significant correlation. However, year of publication [β: − 0.09, (95% CI: − 0.79–0.61, *p* = 0.81)] did not show a statistically significant relationship. In summary, infection from hookworms was more prevalent among children in Amhara region compared to other regions and among SAC compared to PSAC (Table 6).

**Table 6:** Pooled prevalence of species specific Hookworms by region, age and year of publication among Ethiopian children, 2020

Variables	No. of studies	Sample	cases	Prevalence (95% CI)	Heterogeneity		*p *value
					Q	I^2^ (%)	
Region							
Benishangul-Gumuz	1	422	35	8 (6–11%)	–	–	–
Amhara region	32	35,678	6171	16 (13–19%)	5256.02	99.41	*p* < 0.001
Oromia region	15	6763	434	6 (5–8%)	392.85	96.44	*p* < 0.001
South Region	20	8761	950	12 (9–15%)	793.61	97.61	*p* < 0.001
Tigray region	8	5665	144	3 (2–4%)	96.14	92.72	*p* < 0.001
Age							
School	67	53,289	7648	13 (11–15%)	7814.96	99.16	*p* < 0.001
Preschool	8	3740	102	2 (1–3%)	33.50	79.10	*p* < 0.001
Both	1	260	4	2 (1–4%)	–	–	–
Year of publication							
1990–2012	19	11,253	1534	13 (9–15%)	1346.55	98.66	*p* < 0.001
2013–2020	57	46,036	6220	11 (9–13%)	6088.71	99.08	*p* < 0.001
Overall	76	57,289	7754	12 (10–13%)	7920.16	99.05	*p* < 0.001

*CI* Confidence Interval

#### Strongyloides stercoralis

Twenty six studies consisting of 11,748 children have reported that the pooled prevalence of *Strongyloides stercoralis* was 1% (95% CI: 1–2%). The pooled prevalence of *Strongyloides stercoralis* was 3% (95% CI: 1–4%) in Amhara region, 1% (95% CI: 1–2%) in SNNPR, 1% (95% CI: 0–1%) in Oromia region and 0% (95% CI: 0–1%) in Tigray region as shown in Additional file 1: Fig. S7. The prevalence was 1% (95% CI: 1–2%) in SAC. The pooled prevalence of *Strongyloides stercoralis* was 1% (95% CI: 1–2%) in studies done between 1997 and 2012 years and 2% (95% CI: 1–2%) between 2013 and 2020.

A univariate meta-regression between prevalence and regional states showed statistically significant correlation [Β = − 0.30, (95% CI: − 0.56 to − 0.03, *p* = 0.03)] (Additional file 1: Fig. S8). However, year of publication [β: − 0.17, (95% CI: − 0.70 to 1.0, *p* = 0.70)] and age of children [β: − 0.02, (95% CI: − 0.96 to 0.92, *p* = 0.97)] did not show a statistically significant relationship. Therefore, *Strongyloides stercoralis* is more common among children in the Amhara region compared to other regions (Table 7). For further details, the summary of species-specific STHs presented in Table 8.

**Table 7:** Pooled prevalence of species specific *Strongyloides stercoralis* by region, age and year of publication among Ethiopian children, 2020

Variables	No. of studies	Sample	cases	Prevalence (95% CI)	Heterogeneity		*p* value
					**Q**	**I**^**2**^** (%)**	
*Region*							
Amhara region	11	5131	163	3 (1–4%)	116.39	91.41	*p* < 0.001
Oromia region	5	1566	10	1 (0–1%)	2.13	00.00	*p* = 0.71
South Region	7	3149	54	1 (1–2%)	35.77	83.23	*p* < 0.001
Tigray region	3	1902	9	0 (0–1%)	–	–	–
Age							
School	23	10,134	204	1 (1–2%)	150.65	85.40	*p* < 0.001
Preschool	2	1492	30	0 (0–1%)	–	–	–
Both	1	122	2	2 (0–6%)	–	–	–
*Year of publication*							
1990–2012	11	5653	79	1 (1–2%)	141.55	90.11	*p* < 0.001
2013–2020	15	6095	157	2 (1–2%)	6088.71	72.96	*p* < 0.001
Overall	26	11,748	236	1 (1–2%)	179.49	86.07	*p* < 0.001

*CI* Confidence interval

**Table 8:** summary of species-specific pooled prevalence estimates of STHs among Ethiopian children, 2020

Parasites	Number of studies	Sample size	positives	Prevalence (%)	95% CI	Heterogeneity		*p* value
						Q	I^2^ (%)	
*Ascaris lumbricoides*	85	58,234	9339	17	15–19%	8961.94	99.06	*p* < 0.001
*Trichuris trichiura*	76	54,854	3312	6	6–7%	3766.86	98.01	*p* < 0.001
Hookworms	76	57,289	7754	12	10–13%	7920.16	99.05	*p* < 0.001
*Strongyloides stercoralis*	26	11,748	236	1	1–2%	179.49	86.07	*p* < 0.001

*CI* Confidence Interval

#### The intensity of STHs infection

Only 13 out of 88 studies included 5, 676 children reported about intensity of infection of STHs. Low intensity of infection of A*. lumbricoides* was observed in 16% (95% CI: 10–21%), (Additional file 1: Fig. S9) children. Moderate and high intensity of infections of *A. lumbricoides* were observed in 13% (95% CI: 7–19%), (Additional file 1: Fig. S10) and 6% (95% CI: 2–11%), (Additional file 1: Fig. S11) of children, respectively. Low, moderate and high intensity of infections of *T. trichura* were observed in 16% (95% CI: 12–20%), (Additional file 1: fig. S12), 3% (95% CI: 2–4%), (Additional file 1: Fig. S13), 1% (95% CI: 1–2%, (Additional file 1: Fig. S14) children, respectively. This review also showed that low, moderate and high intensity of infections of Hookworms were recorded in 20% (95% CI: 10–29%), (Additional file 1: Fig. S15), 4% (95% CI: 2–6%), (Additional file 1: Fig. S16) and 5% (95% CI: 0–11%), (Additional file 1: Fig. S17) children, respectively.

### Regional distribution of eligible studies and risk zones (RZs) for STHs infections

The highest numbers of studies were reported from Amhara 36 (40.90%) and SNNPR 22(25%). These were followed by the Oromia region 20 (22.7%), Tigray 8 (9.1%), Benishangul-Gumuz region, and Addis Ababa city each with one (1.1%) study. None of the regions is classified as High-Risk Zone (HRZ) according to the world health organization (WHO) risks classification. SNNPR, Amhara, and Oromia regions recorded STH prevalence of 44%, 34%, 31%, respectively and are classified as moderate-risk zones (MRZs) while, the rest of the regions and cities recorded prevalence estimates ranging between 1 and 10% and are classified as Low-Risk Zones (LRZs).

## Discussion

The purpose of the current systematic review and meta-analysis of STHs infections data analysis among Ethiopian children was to measure the impact of the ongoing control and preventive measures in the country and support the efforts undertaken to control and eliminate neglected tropical diseases (NTDs) by nurturing or supplementing useful national epidemiological data. Such studies have the potential to guide concerned bodies to focus their efforts in highly endemic areas. Although several studies have been published from different regions of Ethiopia on STHs with the earliest scientific literature dating back 1990s, the data on STHs infections remains unorganized and scattered. Therefore, organizing and locating information has the potential to inform and develop a comprehensive approach to control STH infections and target highly endemic areas with greater urgency.

The overall pooled estimate of STHs (33%) observed in the present review is in line with the study from south America 27.1% [118], but higher than the study done in Iran (9.48%) [119] and Côte d'Ivoire (19.1%) [120]. The prevalence is lower than studies from Nigeria (54.8%) and reports from other Sub-Sharan African countries (52.4 and 65.8%) [121]. The variation between the findings might be attributed to differences in sensitivity and specificity of diagnostic methodology, environmental factors such as soil moisture, humidity, temperature, and level of participants’ hygiene and sanitation. In addition, our review included more recent surveys that the ongoing MDA and Sustainable water, sanitation, and hygiene (WASH) programs decreased the prevalence of STHs in Ethiopian children unlike the systematic review from Nigeria which included old studies from the year 1985 [121].

Subgroup analysis of the current review also showed that STHs are more common in SNNPR, Amhara, and Oromia regions, although variation among the regional states was not statistically significant (*p* = 0.70). The majority of these infections are related to the low standard of living, poor socioeconomic status, poor personal hygiene, and poor environmental sanitation. The higher prevalence of STHs infection among children in SNNPR, Amhara and Oromia regions might be also related to the high rainfall, forest, and low temperature which favors the survival and transmission of the helminths in these regions. The lowest prevalence in Addis Ababa, the capital city of Ethiopia, might be due to an advanced lifestyle, good personal hygiene, and good quality of life.

Our review suggests that the risk of STH infections has decreased from 44 to 30% in studies conducted between 1997–2012 and 2013–2020 respectively, although the decline is not statistically significant (*p* = 0.45). Prevalence might have declined in some parts due to improvement in living conditions globally and expansion of major deworming efforts, including in Ethiopia. Nevertheless, the increase in population growth in Ethiopia is tremendous and therefore, might have increased the numbers infected and resulted in a slight decline in rate. It is also suggested that the widespread use of monotherapy of antihelminthic for deworming purposes might have facilitated the development of drug resistance and hence, decreased the rate of decline STHs in general and hookworms in particular [122, 123].

If environmental and behavioral conditions are not changed at the same time that the chemotherapy program is being implemented, the prevalence will tend to return to original pretreatment levels through reinfection and therefore, need a holistic approach [3, 123–128]. According to WHO risk categorization, our finding (33%) indicates that MRZ of STHs requires MDA once annually, specifically in SNNPR, Amhara, and Oromia regions.

Concerning the species of STH, *A. lumbricoides* was the predominant species with a prevalence of 17% indicating that about one in six of Ethiopian children is living with Ascariasis. The current prevalence of the parasite is higher than the findings from other countries such as Iran (0.75%) and Srilanka (2.8%) which indicated that indoor and outdoor biotic contamination of the living environment arising partly from improper disposal of human waste, and partly from the integration of the lives of humans and animals of Ethiopian community might account for the still-high rate of the infection in the country. The finding of the current review (17%) is in line with findings from South America (15.6%), studies conducted in the Amhara region, Ethiopia (16.8%), and the overall burden in Sub-Saharan African countries (15%) [129]. However, it was lower than the results from Nigeria (44.6%), Rwanda (38.6%), Uganda (43.5%) [130], Kenya (24.3%) [131], and previous estimates in Ethiopia (37%). The observed differences might be due to variation in some factors putting the population at risk of acquiring STHs such as geographical variations, the lifestyle of the community, soil humidity, and exposure to contaminated environments.

In the current review, the prevalence of *A. lumbricoides* significantly decreased from 25% in 1997–2012 to 14% in 2013–2020 (*p* = 0.006). There was a 49% decline in the prevalence of *A. lumbricoides* observed before the implementation of the MDA program in school children compared to post-MDA. This risk reduction might be related to improved sanitation, access to better water supply, improved personnel hygiene, or the higher efficacy of the available treatments against A lumbricoides [122]. In support of this, a local study conducted on the efficacy of albendazole and Mebendazole indicated that the drugs have 95% efficacy in decreasing the burden of the parasite in Ethiopia [132].

The pooled prevalence of 6% observed for *T. trichiura* was higher than the 1.9% and 3.4% reported from Uganda [119] and Rwanda [121], respectively. The present finding is, however, lower than the reports of the disease burden of Sub-Saharan Africa (13%) [106], Nigeria (18.2%) [110], and Cameroon (15.6%) [118]. This might be due to the geographical variations, the lifestyle of the community, soil humidity, and exposure to contaminated environments. Meta-regression analysis by year of publication revealed that the prevalence of *T. trichiura* decreased from 14% in 1997–2012 to 4% in 2013–2020 (*p* = 0.03). The reason behind the substantial decrease in the prevalence of *T. trichiura* in the country during the study period might be due to the synergistic effect of overall improvements in sanitation, personnel hygiene, and deworming programs.

The finding of the current review showed that the prevalence of hookworms was 12% indicating that the current finding is lower than other studies conducted in Nigeria (32.7%) [121] and Uganda (18.5%) [130]. However, it was higher than studies conducted in Kenya (0.4%) [131], Rwanda (1.8%) [133] and Cameroon (3.9%) [134]. In general, increments of prevalence in our data might be attributed to the re-infection rate, low coverage or unequal distribution of MDA in all regions of the country, level of poverty (walking bare of the foot), and lack of good quality of life. For instance, most Ethiopians are living in the rural area and engaged in agriculture. Engagement in agricultural pursuits remains a common denominator for adult human hookworm infection, which might serve as a reservoir for reinfection of children [128]. Hookworm did not show a significant trend of decrement in prevalence between 1997 and 2012 (13%) as compared to the years between 2013 and 2020. This is, in contrast, to a study conducted in Nepal where the prevalence of Hookworms significantly decreased between the 1990s and 2015[135].

Eliminating STHs as a public health has to go beyond preventive chemotherapy for SAC, as other groups at risk also serve as a reservoir of infection (preschool children and pregnant women, and even adults), which might have resulted in a slight decline again. It is also suggested that the widespread monotherapy of antihelminthic for deworming purposes might have facilitated the development of drug resistance and hence, decreased the rate of decline STHs in general and Hookworms in particular [122, 123].

The strengths of our review include a rigorous search of several databases and other sources to identify eligible studies on the large pediatric population infected by STHs and generate data for policymakers to strengthen or modify the already ongoing control and prevent measures on the place. We also estimated the geographical distribution and identified risk areas that should be prioritized for MDA and other control interventions, which complement global efforts towards the elimination of STHs and other parasitic infections by 2020. In addition, this work also highlighted the need for surveys in areas where data are not available such as the Somalia region, Afar region, Harari, Dire Dawa city, and Gambela regions or scanty (Addis Ababa city and Benishangul-Gumuz region). There are a few limitations of the present meta-analysis. First, It is prudent to interpret the results of this study as 34(38.6%) of the included studies were low quality based on our quality assessment criteria. Second, in almost all of the studies included in this review, single stool sample examinations were used despite multiple stool samples recommendation for standard diagnosis, and therefore, there is a possibility for underestimation of the prevalence. Almost all studies included the current analyses examined the stool specimens for many parasites at a time and the diagnostic performance of such an approach is not known compared to studies that examine solely for STHs, such a diagnostic approach might affect the detection rate and prevalence estimates of STHs infections. Third WHO has recommended the Kato-Katz method as the best and most reliable diagnostic tool with better efficacy, accuracy, and predictive value than other techniques in resource-poor settings [136]. However, only 39.8% of the studies reported the use of the Kato-Katz method or in combination with other methods. Morbidity due to STH infections is a result of worm burden (number of eggs per gram of feces), otherwise called infection intensity. The disease prevalence is commonly combined with the intensity of infection to assess the epidemiological situation for STH infections and to classify communities into transmission categories, which enables the determination of the appropriate strategies for treatment and control [137]. However, only a few studies (13 out of 88) reported the intensity of infection of species-specific STHs, and thus, difficult to reach on definitive conclusion about the intensity of infection of STH in Ethiopia children. Therefore, there is an urgent need for a large-scale study to assess the intensity of infection of STH in children using the sensitive diagnostic tool on a repeated stool sample. Finally, the review protocol has not registered ahead of actual meta-analysis, which could be a source of bias.

## Conclusions

Despite efforts made to reduce, STHs infection is still highly prevalent across the Ethiopian region with some degree of variation. Southern, Amhara, and Oromia regional states carry the highest burden. We observed a decreased prevalence of STHs among Ethiopian children post-MDA compared to preMDA, but the decrease is not statistically significant. *A. lumbricoides* had the highest prevalence among STHs. *A. lumbricoides* and *T. trichiura* were the most prevalent species in the Southern region while hookworms recorded the highest prevalence in the Amhara region. With effort made by the country in eradicating STHs infections, none of the regions in the country is classified as HRZ according to WHO risk classification. Southern, Amhara, and Oromia regions carried the moderate burden and are classified as MRZs, and therefore annual MDA is recommended while, the rest of the regions and city are classified as low-risk zones LRZs. We hope that the results of this study will provide valuable information to policymakers, the National Health Bureau, and other interested bodies, in particular on the endemicity, national and regional distribution, and the prevalence of STHs species in Ethiopia. The high prevalence of STHs observed in this review, underscores the need for better control and prevention strategies in Ethiopia.

## Supplementary Information

**Additional file 1:** Forest plot and meta-regression result of the prevalence of soil-transmitted helminths infections by region and species.

## Data Availability

The datasets used and/or analyzed during the current study are available from the corresponding author on reasonable request.

## Abbreviations

STH: Soil-transmitted Helminth
MRZ: Moderate risk zone
HRZ: High-risk zone
LRZ: Low-risk zone
MDA: Mass Drug Administration
GRADE: Grading of recommendations assessment, development, and evaluation
CI: Confidence interval
PRISMA: Preferred reporting items for systematic reviews and meta-analyses
WHO: World Health Organization
NTDs: Neglected tropical diseases
SAC: School age children
PSAC: Pre-school age children
MeSH: Medical subject headings
WASH: Water, sanitation and hygiene
SNNPR: Southern Nations, Nationalities, and Peoples' Region
