# Supplementary material for: Prevalence of soil-transmitted helminths infections among preschool and school-age children in Ethiopia: a systematic review and meta-analysis
Source: Glob Health Res Policy. 2022 Mar 21;7:9. doi: 10.1186/s41256-022-00239-1 (PMC8935818; doi:10.1186/s41256-022-00239-1)
Supplement: Supplementary file 1 — Additional file 1: Forest plot and meta-regression result of the prevalence of soil-transmitted helminths infections by region and species. [file 41256_2022_239_MOESM1_ESM.docx]

**Supplementary 1**: Forest plot showing the prevalence o**f** A***. lumbricoides*** by region

**Supplementary 2**: Meta regression result of *A.* ***lumbricoides*** by year of publication.

**Supplementary 3:** Forest plot showing prevalence of ***T. trichiura*** by region

**Supplementary 4:**  Meta regression result of ***T. trichiura*** by year of publication

**Supplementary 5:** Forest plot showing the prevalence of hookworm by region

A

B


**Supplementary 6:** Meta regression result of A. the geographic distribution B. the distribution by age of **Hookworms**

**Supplementary 7:** Forest plot showing the prevalence of ***Strongyloides stercoralis*** by region

**Supplementary 8:** Meta regression result of prevalence of ***Strongyloides stercoralis*** by region

**Supplementary 9:** forest plot showing prevalence of low intensity of infection of *A.* ***lumbricoides***

**Supplementary 10:**  forest plot showing prevalence of moderate intensity of infection of *A.* ***lumbricoides***

**Supplementary 11:**  forest plot showing prevalence of high intensity of infection of *A.* ***lumbricoides***

**Supplementary12:**  forest plot showing prevalence of low intensity of infection of ***T. trichiura***

**Supplementary 13:**  forest plot showing prevalence of moderate intensity of infection of ***T. trichiura***

**Supplementary 14:**  forest plot showing prevalence of high intensity of infection of ***T. trichiura***

**Supplementary 15:**  forest plot showing prevalence of low intensity of infection of Hookworms

**Supplementary 16:**  forest plot showing prevalence of moderate intensity of infection of Hookworms

**Supplementary 17:**  forest plot showing prevalence of high intensity of infection of Hookworms
